# Hormonal Responses to Susceptible, Intermediate, and Resistant Interactions in the *Brassica napus*–*Leptosphaeria maculans* Pathosystem

**DOI:** 10.3390/ijms22094714

**Published:** 2021-04-29

**Authors:** Cunchun Yang, W. G. Dilantha Fernando

**Affiliations:** Department of Plant Science, Faculty of Agriculture and Food Science, University of Manitoba, Winnipeg, MB R3T 2N2, Canada; umyang48@myumanitoba.ca

**Keywords:** *Leptosphaeria maculans*, *Brassica napus*, hormone signaling, gene expression, salicylic acid (SA), jasmonic acid (JA), ethylene (ET), defense

## Abstract

Hormone signaling plays a pivotal role in plant–microbe interactions. There are three major phytohormones in plant defense: salicylic acid (SA), jasmonic acid (JA), and ethylene (ET). The activation and trade-off of signaling between these three hormones likely determines the strength of plant defense in response to pathogens. Here, we describe the allocation of hormonal signaling in *Brassica napus* against the fungal pathogen *Leptosphaeria maculans*. Three *B. napus* genotypes (Westar, Surpass400, and 01-23-2-1) were inoculated with two *L. maculans* isolates (H75 8-1 and H77 7-2), subsequently exhibiting three levels of resistance: susceptible, intermediate, and resistant. Quantitative analyses suggest that the early activation of some SA-responsive genes, including *WRKY70* and *NPR1*, contribute to an effective defense against *L. maculans*. The co-expression among factors responding to SA/ET/JA was also observed in the late stage of infection. The results of conjugated SA measurement also support that early SA activation plays a crucial role in durable resistance. Our results demonstrate the relationship between the onset patterns of certain hormone regulators and the effectiveness of the defense of *B. napus* against *L. maculans*.

## 1. Introduction

Plant hormones (or phytohormones) refer to a group of small biomolecules that flow throughout the plant body and play various roles in the physiological processes and signal transduction. Plant defense, as one of these biological processes, involves the co-operation of multiple hormones [1,2]. Among these hormones, salicylic acid (SA), jasmonic acid (JA), and ethylene (ET) are considered to play major roles [1]. Conventional theory from previous studies (in Arabidopsis) suggested that the signals from SA and ET/JA have antagonistic relationships to each other. SA is more effective in defending against biotrophic and hemi-biotrophic pathogens, while ET/JA signaling is more capable of resisting necrotrophic pathogens and herbivorous insects [1,3].

Each hormone has certain responsive factors and signaling pathways, where the responsive pathways of different hormones also have different potential connections, building up an integrated and systemic signaling network in order to cope with various challenges [1,2,3]. *WRKY70* encodes a transcription factor that lies on the node between SA and JA signaling; the up-regulation of *WRKY70* activates SA signaling and suppresses JA signaling [4]. In *Arabidopsis thaliana*, *Coronatine-Insensitive 1* (*COI1*) has been found to regulate JA signaling in root growth, plant defense, and senescence [5]. *Ethylene Insensitive 3* (*EIN3*) encodes a nucleus-localized transcription factor that positively regulates ET signaling [6]. Hormonal pathways eventually induce responsive downstream proteins that have anti-microbial activities. For instance, Pathogenesis-Related Protein 1 (PR1) proteins (encoded by *PR1* genes) are the responsive factors of SA signaling [2], while another *PR* gene—*Pathogenesis-Related Protein 4* (*PR4*)—is activated by ET/JA signaling [7].

*B. napus* has two types of in vivo resistance to *L. maculans*: qualitative and quantitative [8]. Qualitative resistance is triggered by the interaction between the Avr proteins of the pathogen (AvrLm for *L. maculans*) and R proteins from the host (Rlm for *B. napus*). This type of interaction is also called an incompatible interaction (resistance), while an interaction without the Avr–R protein interaction is called a compatible interaction (susceptible). Incompatible interactions trigger a series of rapid and localized host signaling cascades named hypersensitive response (HR), which includes reactive oxygen species (ROS) production, programmed cell death (PCD), and systemic acquired resistance (SAR) [8,9].

Previous studies have suggested that compatible and incompatible interactions may have similar molecular signaling network profiles, including hormonal secretion and signaling. In the *Arabidopsis thaliana*–*Pseudomonas syringae* pathosystem, the expression profiles between compatible and incompatible interactions are similar; however, some genes in the incompatible interaction are activated earlier than in the compatible interaction, which makes the incompatible interaction more robust [10,11]. By studying the pathosystem between *Arabidopsis thaliana* and *Alternaria brassicicola*/*Pseudomonas syringae* pv. tomato DC3000, it has been shown that the R-protein resistance activates the hormone-regulated factors that are able to defend against both biotrophs and necrotrophs [11]. Moreover, studies of the gene-for-gene interaction in the *B. napus*–*L. maculans* pathosystem have also observed the early induction of SA/JA responsive factors, indicating the importance of those genes in the incompatible interaction [12,13]. Plants have developed a huge network of hormonal signaling pathways to cope with pathogenic invasion. Among plant hormones, salicylic acid (SA), jasmonic acid (JA), and ethylene (ET) are the three major phytohormones released in response to plant pathogens [2,3,14]. Therefore, investigating the signaling and interactions among these three hormones becomes necessary to understand the three selected *B. napus* genotypes responded differently to *L. maculans*.

In this study, we selected three *B. napus* genotypes to be inoculated with two *L. maculans* isolates, such that the host cotyledons were able to produce three typical disease severities: susceptible, intermediate, and resistant phenotypes. The goal of this research is to find the connection between the *B. napus* defense and the hormonal signaling, we aimed to find which types of the hormonal regulation (in quantity and onset patterns) are optimal for *B. napus* to understand distinct ways of regulation of the hormonal signaling among the three levels of interaction (i.e., susceptible, intermediate, and resistant). The expression levels of several genes, which are crucial for hormonal–responsive defense, were analyzed in the aspects of both quantity and onset pattern; those analyses help to explain the relationship between hormone signaling and disease severity/host resistance.

## 2. Results

### 2.1. Distinct Levels of Disease Severities from the B. napus Cotyledons with Different Inoculation Pairs

**Figure 1:**
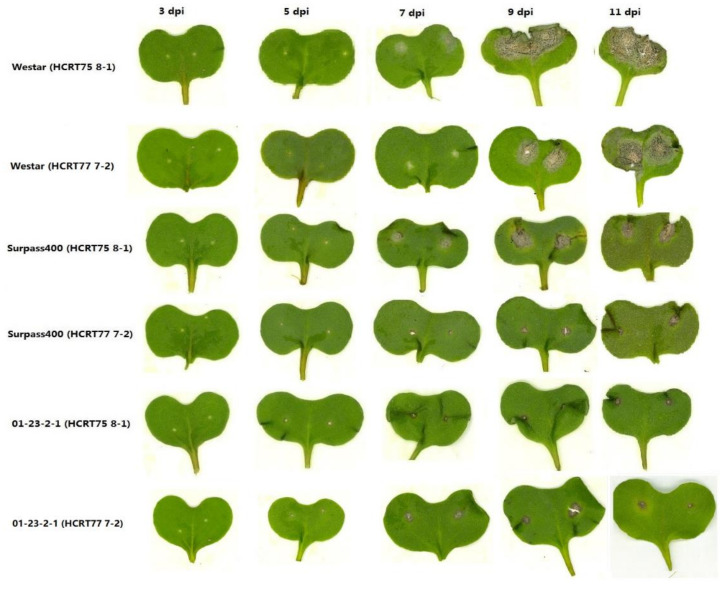
Lesion development on six pairs of *B. napus* cotyledons inoculated with *L. maculans* isolate: Westar—HCRT75 8-1/HCRT 77 7-2, Surpass400—HCRT75 8-1/HCRT77 7-2, and 01-23-2-1—HCRT75 8-1/HCRT 77 7-2 at 3, 5, 7, 9, and 11 days post-inoculation (dpi).

To obtain *B. napus* cotyledons with various levels of disease severity, three *B. napus* genotypes (Westar, Surpass400, and 01-23-2-1) were selected for inoculation with two *L. maculans* isolates (HCRT75 8-1 and HCRT77 7-2; the term “HCRT” will be shortened to “H” for the rest of the article). For the selected *B. napus* cultivars, Westar, which has no *Rlm* genes, was regarded as a typical example for susceptible phenotypes, Surpass400 (*BLMR1*/*LepR3* and *BLMR2*/*LepR2*) as intermediate and resistant phenotypes, and 01-23-2-1 (*Rlm7*) as resistant phenotypes. Isolate H75 8-1 exhibited a compatible interaction (susceptible) with Westar, an intermediate incompatible interaction (intermediate resistant) with Surpass400, and an incompatible interaction (resistant) with 01-23-2-1 (Figure 1), while isolate H77 7-2 exhibited a resistant phenotype with Surpass400 (Figure 1) and the same phenotypes as H75 8-1 with Westar and 01-23-2-1. Westar had a compatible interaction, as it had no *Rlm* genes, while the intermediate resistance for Surpass400 inoculated with H75 8-1 was due to the interaction between *AvrLepR2* and *BLMR2*/*LepR2* (Surpass400: *BLMR1*/*LepR3* and *BLMR2*/*LepR2*) [15]. The incompatible interaction between Surpass400 and H77 7-2 was triggered by the interaction between *LepR3*/*BLMR1* and *AvrLm1*, as both *Rlm1* and *LepR3*/*BLMR1* recognize *AvrLm1* [16,17], while the resistance from 01-23-2-1 against HCRT75 8-1/HCRT77 7-2 was caused by the recognition of *AvrLm7* by *Rlm7* in 01-23-2-1.

**Figure 2:**
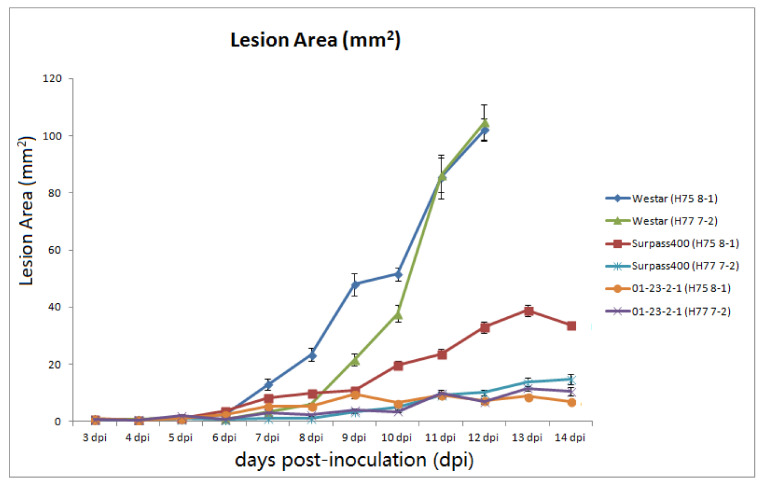
Changes in lesion size (mm^2^) from 3 to 14 dpi in Westar–H75 8-1 (blue curve), Surpass400–H75 8-1, (red curve), Surpass400–H77 7-2 (light green curve), and 01-23-2-1–H75 8-1 (purple curve). The lesion sizes were calculated as the average from the cotyledons of 20 plants (each genotype at each time point).

As shown in Figure 1, the emergence of distinct phenotypes among six sets of host–pathogen combinations did not appear until 5 dpi. From 5 dpi, Westar started to develop lesions at the inoculation sites, while brownish lesions emerged on Surpass400 and 01-23-2-1 cotyledons; in the case of both H75 8-1 and H77 7-2, the HR phenotype (brownish lesions) appeared at 5 dpi. To show the development of lesions in a numerical way, the lesion area was measured for each genotype–isolate pair from 3 to 14 dpi. As the phenotypes from all inoculation pairs emerged at 5 dpi, the lesion size at 3 dpi was set as zero. As shown in Figure 2, Westar–H75 8-1 displayed a rapid development of lesions from 7 dpi and the cotyledons collapsed at 11 dpi, due to the massive fungal colonization. Both Surpass400–H77 7-2 and 01-23-2-1–H75 8-1/–H77 7-2 had slowly increasing lesion areas (Figure 1 and Figure 2). Surpass400–H75 8-1 displayed the gradual development of HR necrotic lesions (brownish lesions) and reached a large size at 11 dpi; however, the plant was still viable and exhibited HR phenotype, this is called the intermediate resistance [15,16].

### 2.2. Fungal Development of L. maculans Isolates from Compatible, Intermediate, and Incompatible Interactions

**Figure 3:**
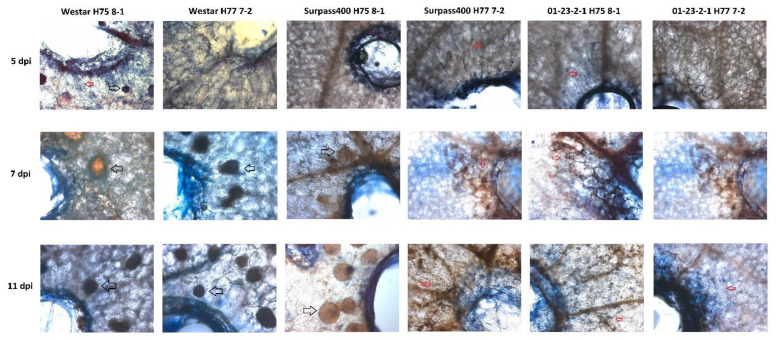
Fungal growth and development on the cotyledons of *B. napus* cv. Westar, Surpass400, and 01-23-2-1 inoculated with *L. maculans* isolates H75 8-1/H77 7-2, as shown by trypan blue staining. Hyphae (red arrows) started to grow on the cotyledon tissue at 5 dpi. The compatible (Westar–H75 8-1/–H77 7-2) and intermediate incompatible (Surpass400–H75 8-1) interactions allowed for the formation of pycnidia (black hollow arrows) at 7 dpi and the fungal tissues formed pycnidia at only 11 dpi. The incompatible interactions (Surpass400–H77 7-2 and 01-23-2-1–H75 8-1/–H77 7-2) did not have pycnidia and only few hyphae grew up to 7 dpi and 11 dpi. The images were taken at 100× magnification.

The presentation of microscopic views of infected cotyledons started at 5 dpi, when the hyphal development (compatible) and necrotic lesions (incompatible) were visible in the microscope (Figure 3). Li et al. (2008) [18] demonstrated hyphal development in intercellular spaces at 5 dpi. At 7 dpi, the three types of severity exhibited distinct patterns of fungal development on the host tissues. On the leaves of oilseeds, *L. maculans* is a fungus that starts its growth as a biotrophic pathogen; after several days of infection, the fungus reaches its necrotrophic stage and fruiting bodies (pycnidia) are formed [19]. As shown in Figure 3, isolates H75 8-1 and H77 7-2 initiated the necrotrophic stage at 7 dpi on susceptible Westar and intermediate Surpass400 (infected by H75 8-1 only), growing pycnidia on the cotyledon tissues on the same day; meanwhile, Surpass400–H77 7-2 and 01-23-2-1–H75 8-1/–H77 7-2 showed few to no pycnidia at the same time point, as the fungal cells on those cotyledons were still in the biotrophic stage (i.e., hyphae only).

For susceptible cotyledons (Westar–H75 8-1/–H77 7-2), the formation of pycnidia occurred at 7 dpi and became dominant at 11 dpi, while 01-23-2-1 did not have any pycnidia, and few hyphae emerged at 7 dpi and 11 dpi after inoculation with H75 8-1 or H77 7-2. For the intermediate Surpass400–H75 8-1 cotyledons, the symptoms lay between susceptible and incompatible interaction phenotypes, the production of pycnidia was, somehow, restricted within the region of necrotic lesions.

To sum up, the different *B. napus* genotypes exhibited distinct responses towards *L. maculans* isolates. For susceptible responses (Westar–H75 8-1/–H77 7-2), apparent hyphal development started at 5 dpi, and the fungus transited to necrotrophic stage at 7 dpi, with the formation of pycnidia; subsequent development was the enhancement of what happened at 7 dpi. Nevertheless, Surpass400 and 01-23-2-1, as more resistant genotypes, displayed delayed fungal development compared with Westar, with the intermediate response from Surpass400–H75 8-1 exhibiting limited pycnidia formation, and 01-23-2-1 had little hyphal development throughout the timeline of observation.

### 2.3. Gene Expression Analysis in Hormone Signaling

The characterization of hormonal signals pathways started with quantitative analyses of the genes responsible for the biosynthesis of the three phytohormones (SA, JA, and ET). To achieve this goal, three hormonal biosynthetic genes were chosen: *ICS1*, *AOS*, and *ACO1*. *ICS1* encodes an enzyme called isochorismate synthase 1, which is involved in salicylic acid biosynthesis [20]. *AOS* encodes an enzyme called allene oxide synthase, which is an enzyme involved in the JA biosynthetic pathway and the octadecanoid pathway [21]. *ACO1* encodes an enzyme called 1-aminocyclopropane-1-carboxylate oxidase 1, which is involved in ethylene biosynthesis in different situations [22]. We analyzed the expression of these three biosynthetic genes in both *L. maculans*-inoculated (H75 8-1 and H77 7-2) and water-inoculated cotyledons at multiple time points after inoculation. The expression levels of the genes from water-inoculated cotyledons were normalized to the level of “1”, in order to find the differential expression levels between control and inoculated cotyledons at each time point, which indicated how the hormonal signals were modulated, when encountering fungal infection. By analyzing the three genes (*ICS1*, *AOS*, and *ACO1*) with regard to the production of SA, JA, and ET, the temporal pattern of biosynthesis-related genes was found to be distinct to the cotyledons among three genotypes.

**Figure 4:**
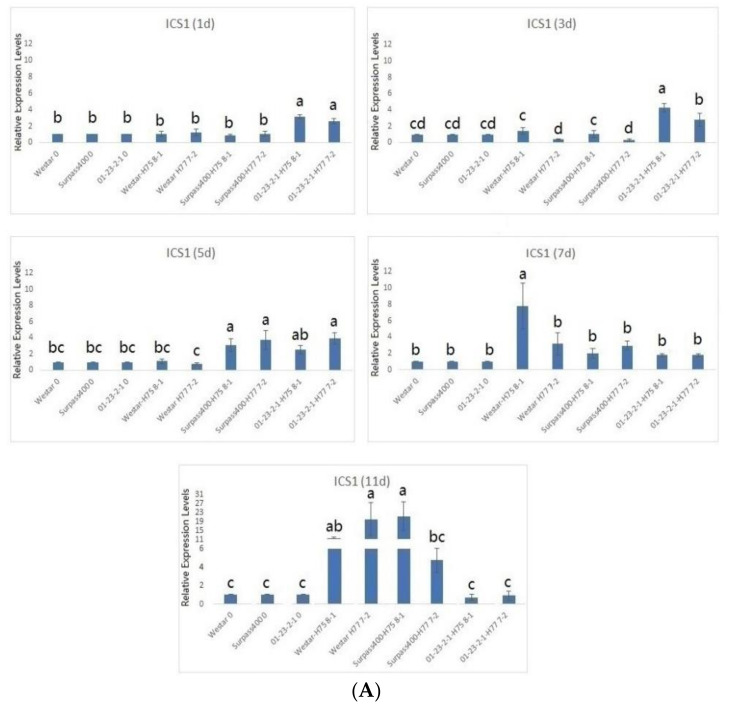

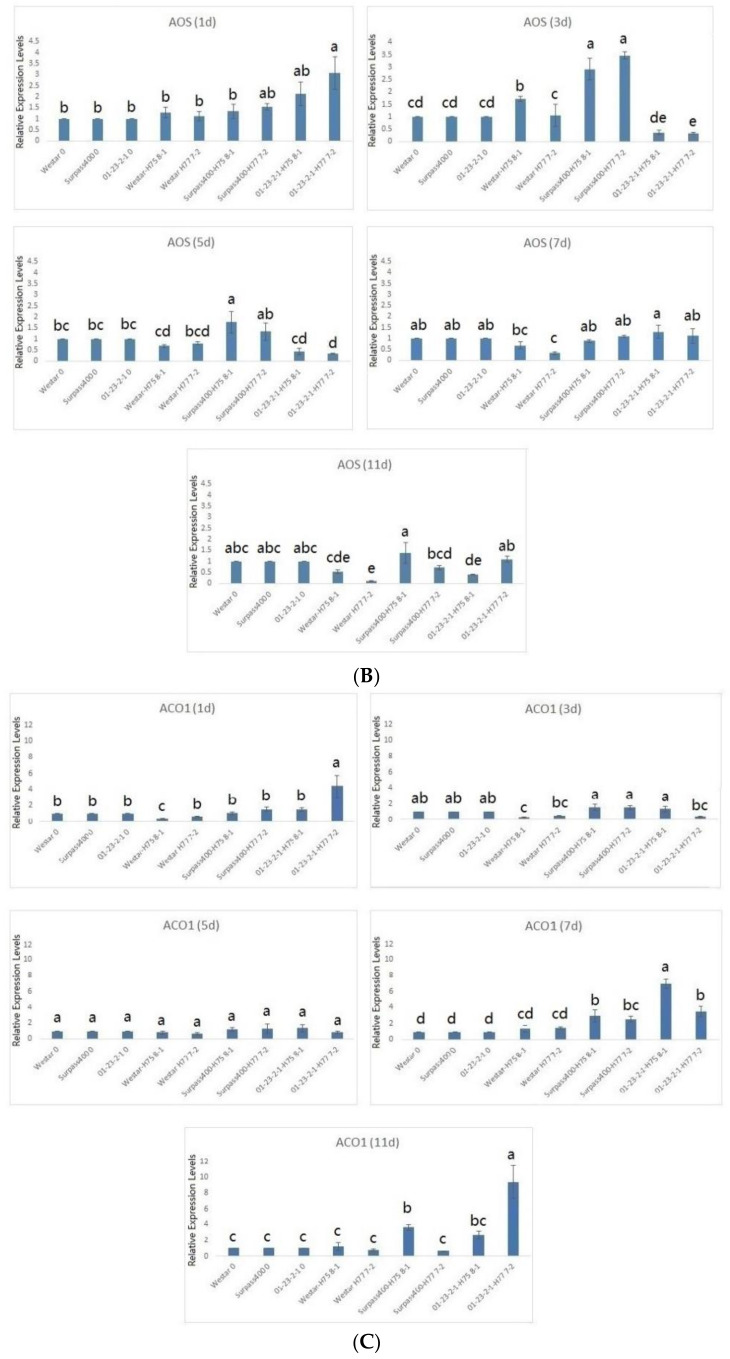
Gene expression in hormonal biosynthesis (ICS1, AOS, and ACO1): the levels of the bars are the expression levels from the inoculated cotyledons (inoculated by H75 8-1 and H77 7-2), compared to the cotyledons inoculated with water (assuming that the expression of each studied gene in the cotyledons inoculated with water is 1). Error bars represent standard error of the mean. For time point, different lowercase letters suggest the significant differences among mean values (Fisher’s least significant difference; *p* < 0.05). The results are based on three replicates in three independent experiments (**A**–**C**).

As shown in Figure 4, the activation of *ICS1* and *AOS* from Surpass400 and 01-23-2-1 were earlier than in Westar; for Surpass400, the earliest timepoint of significantly higher expression of *ICS1* and *AOS* started at 5 and 3 dpi, respectively; however, Westar had lower expression of these two genes at the same timepoints. For 01-23-2-1 cotyledons, all three biosynthetic genes showed earlier expression, as early as 1 dpi. *ICS1* in 01-23-2-1 (both inoculated by H75 8-1 and H77 7-2) showed higher levels of expression at all early time points (i.e., 1, 3, and 5 dpi). Surpass400 – H75 8-1 and 01-23-2-1 displayed late up-regulation of *ACO1* (i.e., 7 and 11 dpi) whilst Westar did not have apparent activation throughout infection. Altogether, the intermediate and resistant genotypes have distinct transcriptional programming compared with the susceptible one, featured by the early activation of SA/JA biosynthetic marker genes *ICS1* and *AOS1*.

### 2.4. The Potential Relationship between Hormonal Biosynthesis and the Regulatory Patterns of Hormonal Signals throughout the B. napus and L. maculans Interaction

**Figure 5:**
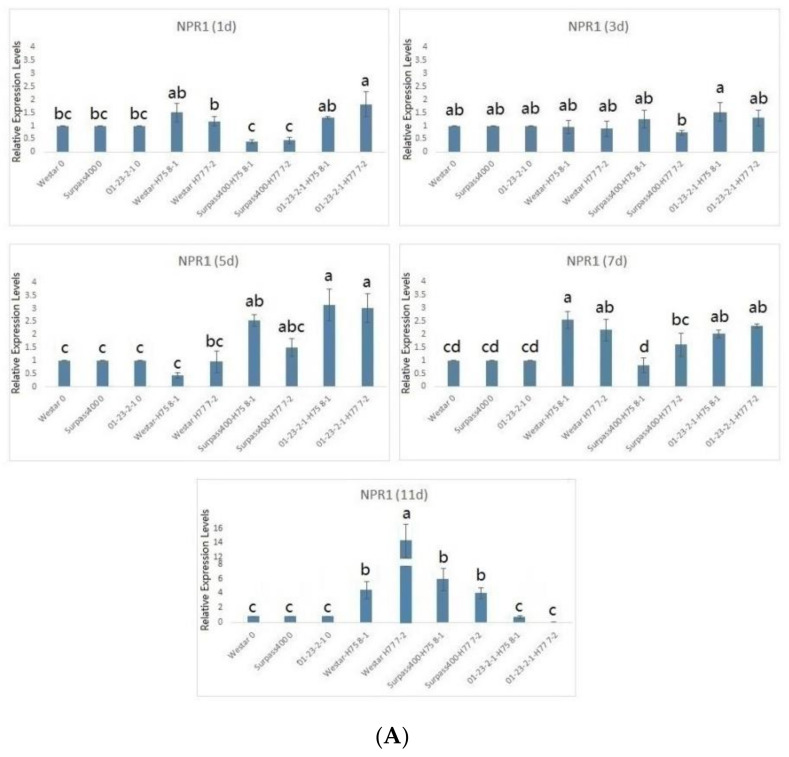

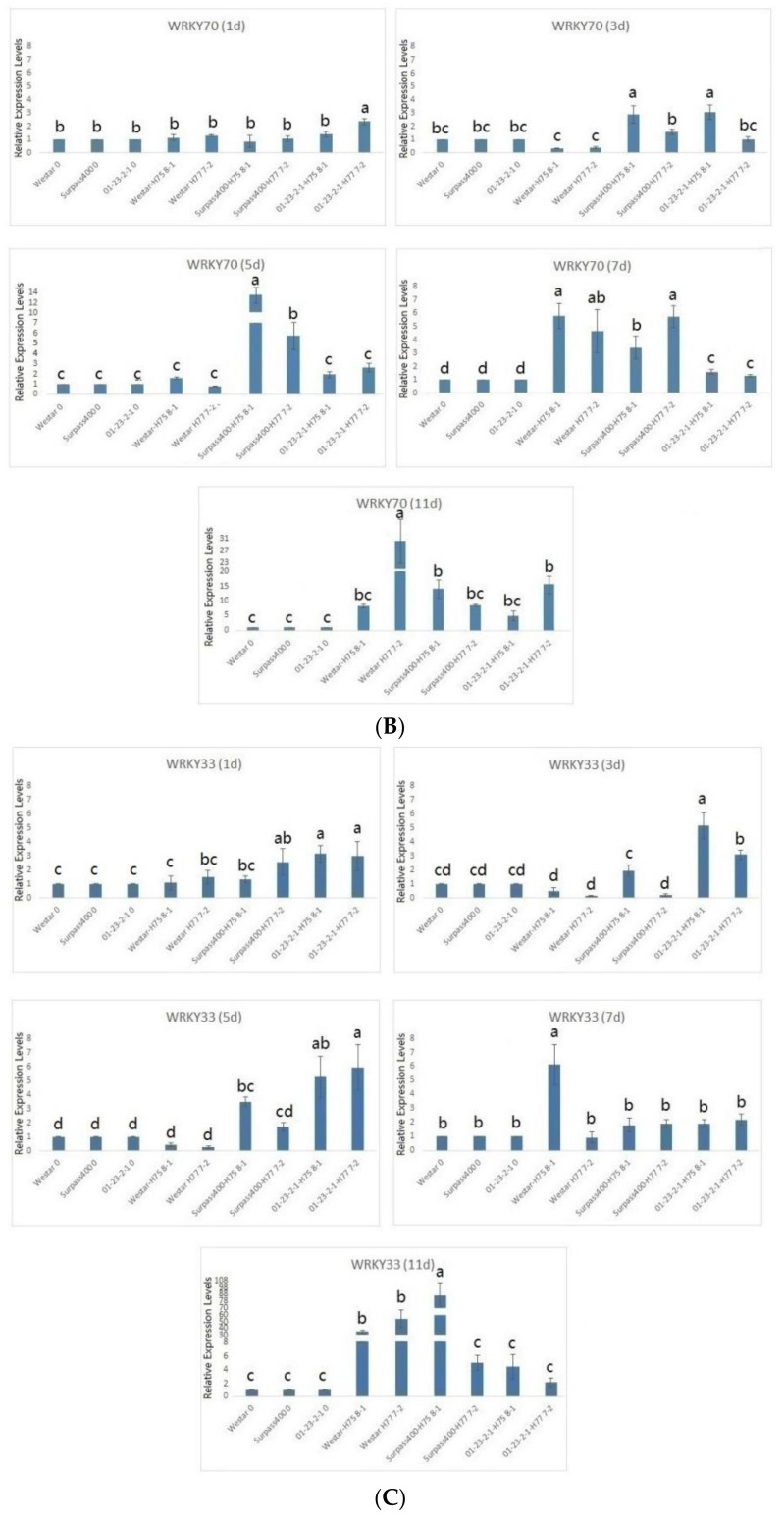

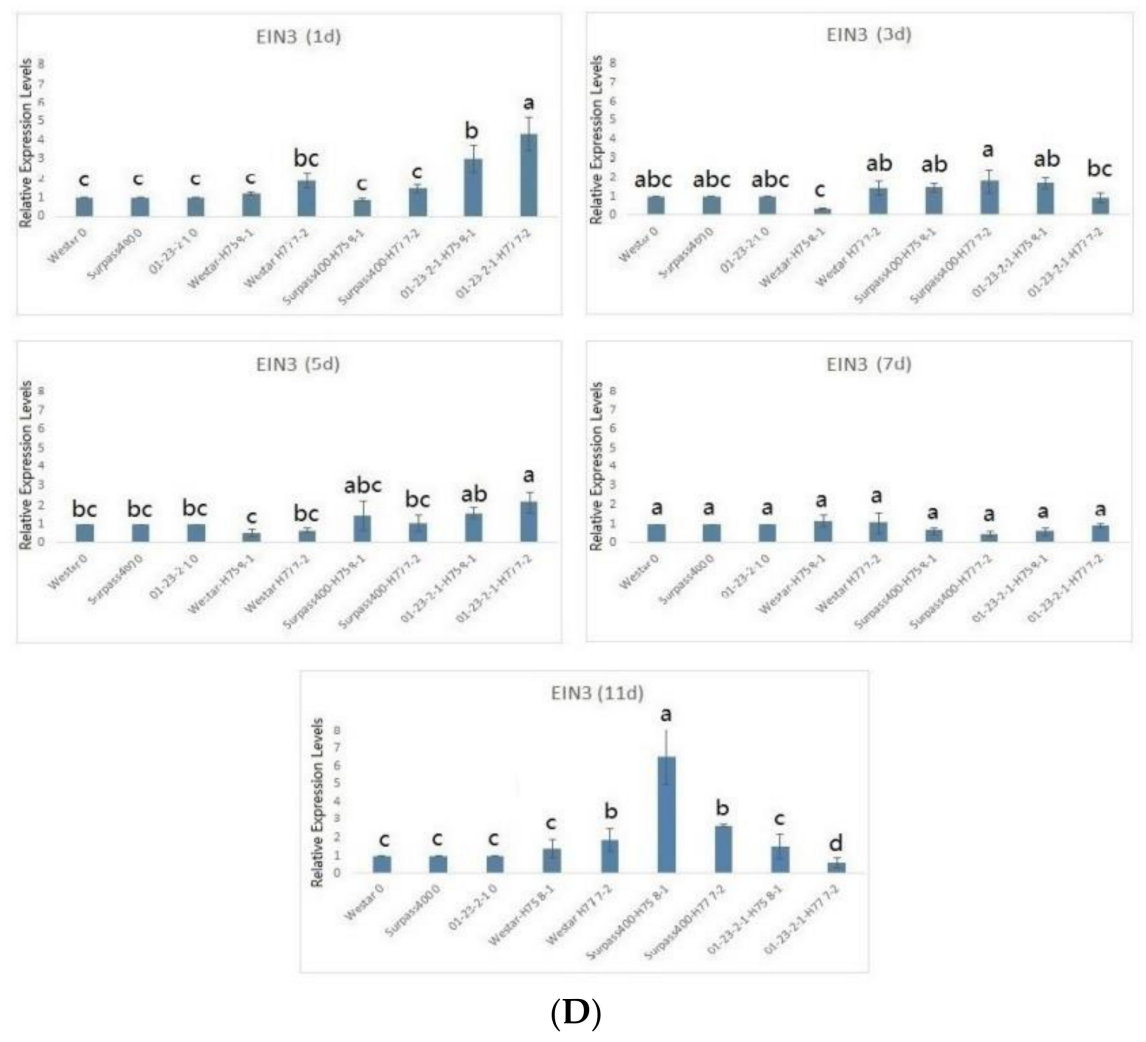
Gene expression in regulation of hormonal signals (NPR1, WRKY70, WRKY33, and EIN3): the levels of the bars are the expression levels in the inoculated cotyledons (inoculated by H75 8-1 and H77 7-2), compared to the cotyledons inoculated with water (assuming that the expression of each studied gene in the cotyledons inoculated with water is 1). Error bars represent standard error of the mean. For time point, different lowercase letters suggest the significant differences among mean values (Fisher’s least significant difference; *p* < 0.05). The results are based on three replicates in three independent experiments (**A**–**D**).

Surpass400–H75 8-1 and 01-23-2-1 had earlier induction of *NPR1* and *WRKY70*, compared with Westar. For *NPR1*, Surpass400 (H75 8-1) and 01-23-2-1 had pronounced expression from 5 dpi; while, for Westar, this pattern did not occur until 7 dpi. For the downstream factor *WRKY70*, Surpass400 and 01-23-2-1 genotypes displayed similar trends, suggesting that the intermediate and resistant cotyledons had earlier SA-related responses (Figure 5). Surprisingly, Surpass400 and 01-23-2-1 also had early activation of ET/JA responsive factor *WRKY33*: both of them induced this gene at 1 dpi, and 01-23-2-1 also had high expression at 3 dpi. Moreover, Surpass400 and 01-23-2-1 also tended to have stronger expressions of *EIN3* than Westar at 3 and 5 dpi; 01-23-2-1 showed high expression of this gene at 1 dpi. In Westar cotyledons (inoculated by both H75 8-1 and H77 7-2), the defense genes started to induce at 7 dpi and reached high levels at 11 dpi. For Westar cotyledons, 7 and 11 dpi are the timepoints when the fungus formed pycnidia and transited into the necrotrophic stage, respectively. The lesions on the infected tissues quickly developed (as shown in Figure 1); therefore, the high levels of defense genes reflected the non-HR-related responses against this deteriorating situation.

**Figure 6:**
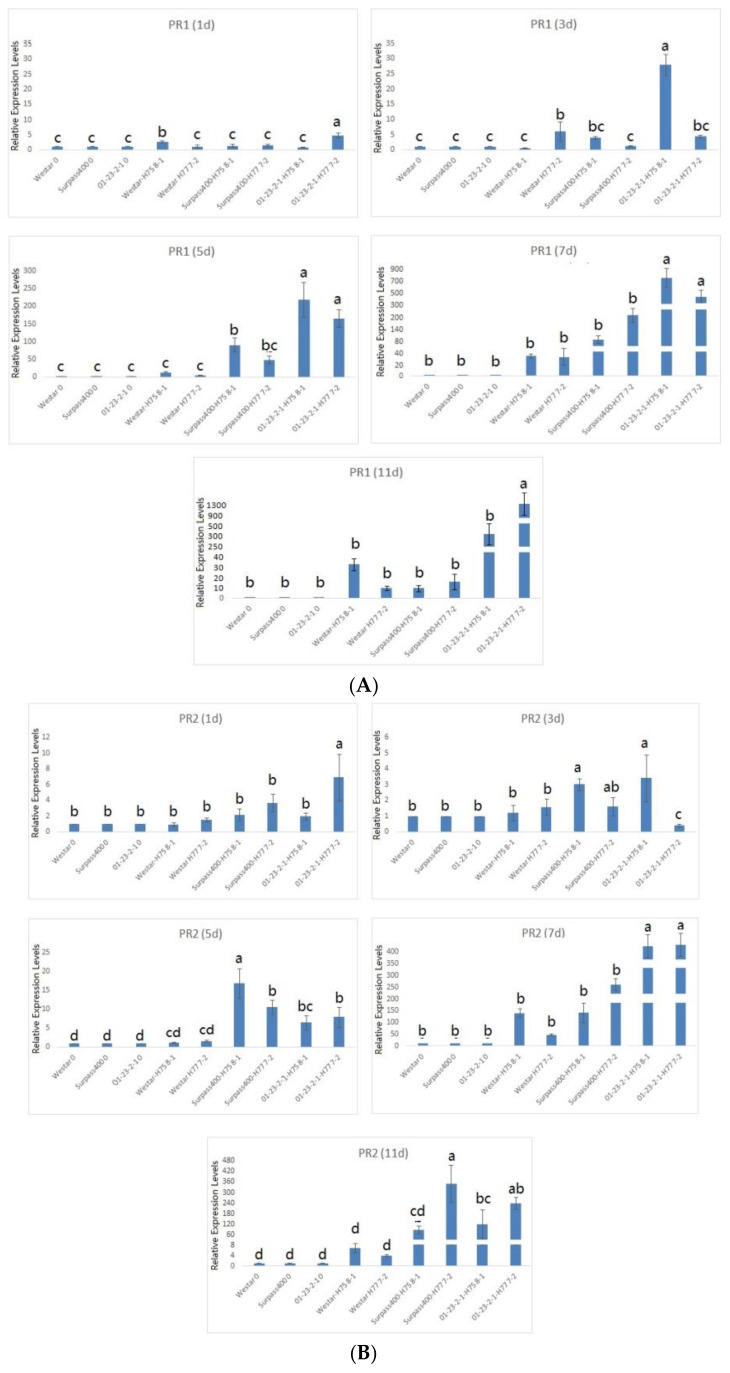

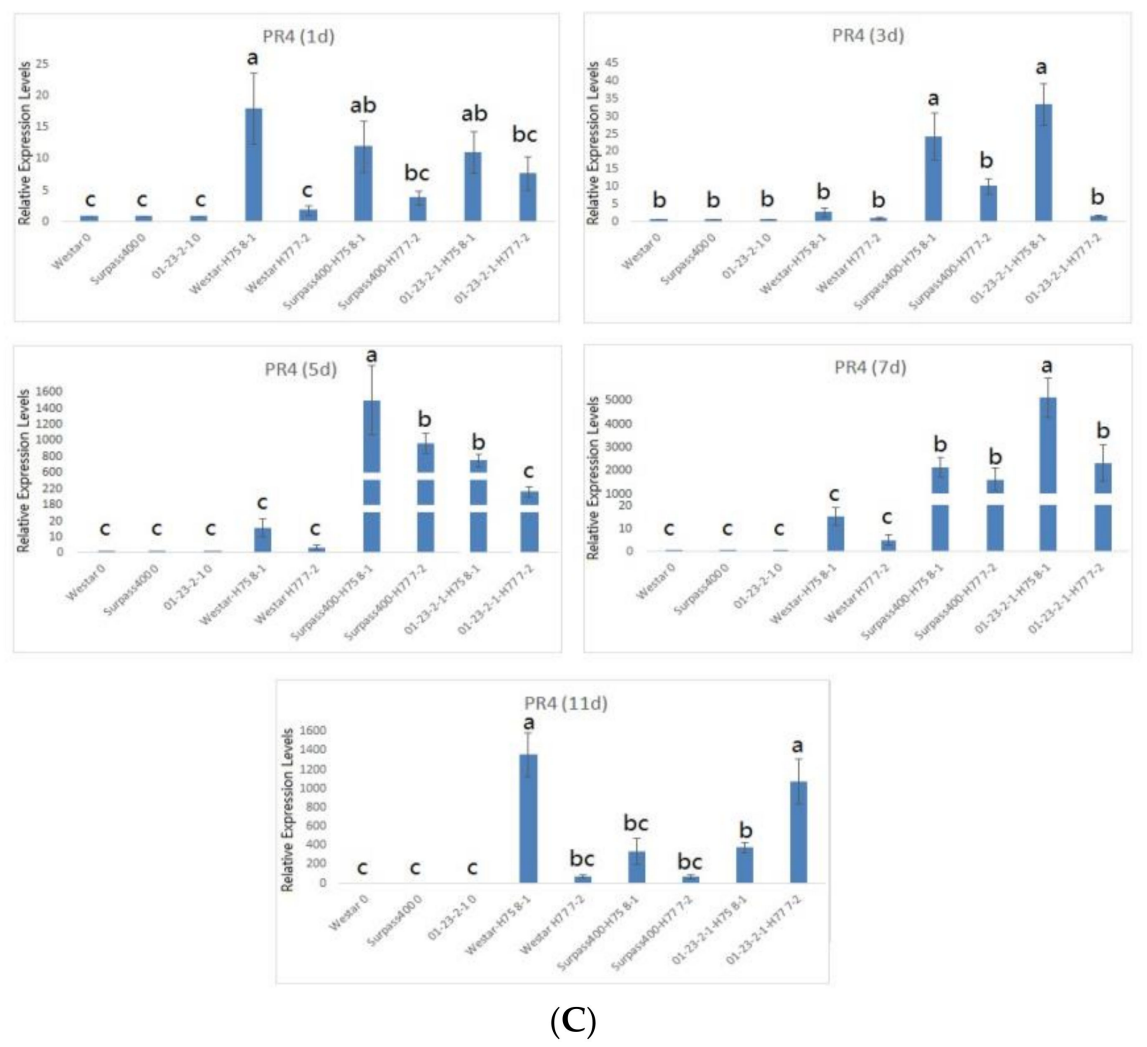
Gene expression of downstream proteins from hormonal signaling (*PR1*, *PR2*, and *PR4*). The levels of the bars are the expression levels from the inoculated cotyledons (inoculated by H75 8-1 and H77 7-2), compared to the cotyledons inoculated with water (assuming that the expression of each studied gene in the cotyledons inoculated with water is 1). Error bars represent standard error of the mean. For time point, different lowercase letters suggest the significant differences among mean values (Fisher’s least significant difference; *p* < 0.05). The results are based on three replicates in three independent experiments (**A**–**C**).

Surpass400 and 01-23-2-1 started to induce high expression of *PR1*, *PR2*, and *PR4* at 5 dpi, while, for Westar (H75 8-1), the massive induction of *PR4* started at 11 dpi (Figure 6). WRKY70, as an SA regulator, positively regulates the expression of pathogenesis-related 1 (PR1) proteins [2]. It seems that the studied transcription factors had somewhat synchronization with the studied *PR* genes; as such, both Surpass400 and 01-23-2-1 showed the activation of regulators (*WRKY70* and *WRKY33*) and *PR* genes (*PR1*, *2*, and *4*) at 5 dpi. On the other hand, there were also discrepancies between transcription factors and downstream *PR* genes. For example, 01-23-2-1 did not show the high induction of *WRKY33* at 7 and 11 dpi, but *PR4* was still very high at the same time points. These results reflect the potential influences of upstream signaling upon the downstream proteins in plant defense; besides, there may have been other factors affecting the expression of downstream proteins.

**Figure 7:**
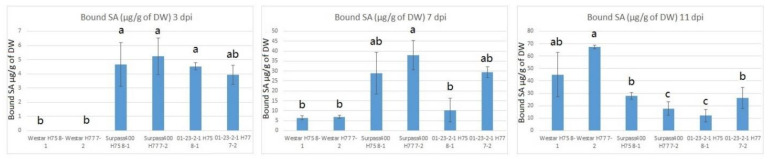
Amount of bound salicylic acid (µg/g of dry weight (DW)) in Westar/Surpass400/01-23-2-1 inoculated with isolates H75 8-1/H77 7-2 at 3, 7, and 11 dpi. Conjugated salicylic acids were hydrolyzed by HCl first, in order to free SA for measurement by HPLC–fluorescence. Error bars represent standard error of the mean. For time point, different lowercase letters suggest the significant differences among mean values (Fisher’s least significant difference; *p* < 0.05). The results are based on three replicates in three independent experiments.

As free SA can be toxic for the living plant, SA signaling induces electrolyte leakage, oxidative burst, and cell death [5,23,24]. As shown in Figure 7, in the intermediate and resistant cotyledons from Surpass400 and 01-23-2-1 genotypes, the presence of bound SA was detected as early as 3 dpi; while it was not detected in the two Westar sample pairs (H75 8-1 and H77 7-2). However, Westar showed later accumulation of SA (at 11 dpi), at which point its levels exceeded those in Surpass400 and 01-23-2-1 cotyledons at the same time point.

## 3. Discussion

Certain hormonal-related factors displayed earlier activation from in intermediate and resistant cases, while susceptible *B. napus* possessed distinct onset patterns of hormonal signal responses. The study suggested that the timing of gene activation might be important to trigger the effective hindrance of fungal growth and development; this type of signal transduction seems to be correlated with the manipulation of fungal development by the host.

### 3.1. The Fungal Development of L. maculans Was Hindered Due to the Host Resistance

According to a study of the susceptible adult leaves of *B. napus* (cv. Westar), the intercellular development of fungal hyphae was observed from the microscope as early as 5 dpi, massive hyphal development throughout the mesophyll was initiated at 7 dpi, and finally, pycnidia were produced on the dead tissues after 11 or 12 dpi [18].

In the case of the Westar–H75 8-1/–H77 7-2 cotyledons, fungal development followed the regular lifecycle of hemi-biotrophic fungus, in which the fungus starts its biotrophic stage from 7 dpi by spreading hyphae, in order to absorb nutrients from the living tissues. Then, it shifted into the necrotrophic stage by producing pycnidia. On the other hand, the incompatible interactions in 01-23-2-1 and Surpass400 restricted the growth and slowed the development of the fungus. One of the associated mechanisms is to induce regional cell death, which creates necrotic lesions on the tissues; this mechanism causes inhibitive growth conditions for biotrophic pathogens, which need living tissues to exploit nutrients [25,26]. On the other hand, Surpass400 and 01-23-2-1 displayed some inconsistency in the further development of necrotic lesions, suggesting unequal intrinsic signaling among the different forms of incompatible interactions. The Surpass400–H75 8-1 combination triggered the gene-for-gene interaction between AvrLmS/AvrLepR2 and RlmS/BLMR2, while that in Surpass400–H77 7-2 was between AvrLm1 and LepR3/BLMR1, and those in 01-23-2-1–H75 8-1/–H77 7-2 were between AvrLm 4-7 and Rlm7 [17,27]. These different types of incompatible interactions may have caused distinct defense signaling network patterns. Therefore, some different onset patterns among the three interactions (i.e., Surpass400–H77 7-2 and 01-23-2-1–H75 8-1/–H77 7-2) may have been due to the different mechanisms of AvrLm–Rlm interactions and/or the subsequent signaling cascades. Surpass400 was remarkable, due to the presence of identified *R* genes *LepR3*/*BLMR1* and *BLMR2*/*LepR2*, associated with these two genes [15,16,28]. *AvrLmS*/*AvrLepR2* was considered as an independent *AvrLm* gene, conferring HR by interacting with *RlmS* [28,29], and the intermediate *R* genes in Surpass400 (*LepR3*/*BLMR1* and *RlmS*/BLMR2/*LepR2*) worked co-operatively with major *Rlm* genes, but also functioned independently of those major genes [16,28].

### 3.2. Fine-Tuning of Hormonal Signals in B. napus Is Able to Resist to L. maculans by Controlling Its Developmental Stages

As there was no apparent hyphal development in all six inoculation pairs before 5 dpi, the differential expression of some of the genes at 1, 3, and 5 dpi implicated that the three genotypes possessed unequal priming response strengths, which were linked to the ability of early sensing of fungal invasion and the anticipated release of defense signals. Studies have revealed that the intrinsic signaling before the emergence of symptoms determines further trends of the host–microbe interaction [12,26]. During the biotrophic stage of *L. maculans*, small secreted proteins (SSPs), including AvrLm proteins, are released into the intercellular space and cytoplasm. The recognition of *L. maculans* AvrLm proteins by *B. napus* Rlm proteins is able to trigger early defense responses [12,26].

During plant defense, the biosynthesis of each of the hormones triggers their responsive transcription factors to activate the downstream genes responsible for curtailing the spread of the disease. Hormonal transcription factors are more downstream proteins, following the activation of MAPKs and biosynthetic enzymes. These factors are triggered by the hormone molecules and impact the expression of some anti-microbial elements, in order to effectively stop further invasion of the pathogens.

### 3.3. The Early Activation of SA-Related Factors (from 1 to 7 dpi) Was One of the Common Features of the Intermediate and Resistant Cotyledons

Generally, Surpass400 and 01-23-2-1 had SA-responsive factors (*ICS1*, *NPR1*, *WRKY70*, *PR1*, and *PR2*) expression higher than that in mock inoculations before 7 dpi, while Westar activated the same set of genes at 11 dpi. SA- and JA-related factors play pivotal roles in plant defense, including HR. *NON-EXPRESSOR OF PR1* (*NPR1*) lies on the node between SA- and JA-dependent defensive signaling, ET modulated the role of *NPR1* to buffer SA and JA signaling, NPR1 positively regulated SA-related defense and negatively regulated JA-related defense, and ET controlled *NPR1* by its responsive factor *ETHYLENE-INSENSITIVE PROTEIN 2* (*EIN2*). NPR1 may also be involved in the full-scale expression of one *WRKY* gene, *WRKY70*, the over-expression of constitutive resistance to some disease by constitutive SA defensive signals, while suppression of *WRKY70* showed increased JA-dependent signals. *WRKY70* encodes a transcription factor that positively regulates SA-related signaling; the over-expression of *WRKY70* also triggers the constitutive expression of *PR1* [22]. *Ethylene-Insensitive 3 (EIN3*) encodes an ethylene-responsive transcription factor; constant ET signaling has been observed as a result of the over-expression of *EIN3* [30].

Becker et al. (2017) [12] also observed the early induction of *ICS1* and *PR1* at 3 dpi in the case of incompatible interaction (AvrLepR1–LepR1), indicating that the early activation of those genes correlates with effective resistance. *PR1* was found to be one of the components and activators of SAR [5]. SAR has defense activity in planta, which is triggered by the primary infection; plant cells secrete mobile substances throughout the plant body in order to prevent secondary infection from the pathogens. Those molecules include many defense-related molecules/proteins, such as PR1 proteins and beta-glucanase (PR2) [31]. *PR2* (also known as *BGL2*) encodes an enzyme called beta-1,3-glucanase, which is also up-regulated following SA accumulation [32]. The PR4, also known as Helvin-Like Protein, HEL protein is regulated by ET-/JA-responsive transcription factors [2]; the PR4 protein is a chitinase that is able to degrade fungal cell walls [14]. The activation of *PR4* indicated the induction of the ET/JA signaling pathways, which are usually responsible for the defense against necrotrophic pathogens [1].

As the fungal development of *L. maculans* (as a hemi-biotroph) initially starts with the biotrophic stage, in this study, around 7 dpi was the transitive time point between biotrophic and necrotrophic phases when colonizing the susceptible *B. napus* genotype (Figure 1). The early activation of SA-responsive factors in Surpass400 and 01-23-2-1 suggested that resistant *B. napus* genotypes are able to effectively slow down the lifecycle of the pathogen and the associated SAR reinforced the defense throughout the plant body; therefore, these two genotypes were able to hinder the fungal development during biotrophic stage. However, the susceptible genotype Westar, after 7 dpi, started to induce hormonal-related defense genes, as the other two genotypes did from 1 to 5 dpi. The late activation of defense genes might be due to the massive colonization during necrotrophic stage, at this stage, the host was barely able to stop the infection, since the amount of fungal load (mycelia) was too large.

Moreover, bound SA measurement also reflected the priming of SA activation, in agreement with the qPCR results (Figure 4 and Figure 5). Usually, SA is synthesized in the chloroplast and transported to the cytosol, where some SA molecules are transformed into bound versions and the inactive bound SA molecules are subsequently displaced in the vacuole for inactive storage [5]. Salicylic acid glucoside (SAG) is one of the derivatives of conjugated salicylic acid (glucolysated form). SAG becomes a slow inducer of SAR and a storage molecule to form free SA. Both SA and SAG play roles in abiotic/biotic stresses, but SAG is a safer and slower agent for oxidative burst and Ca^2+^ leakage [24]. In *Brassica napus*, infection by *Verticillium longisporum* caused the accumulation of SA and SAG from the xylem sap [33]. Stored SAG is able to release SA by hydrolysis to induce oxidative burst and Ca^2+^ leakage for disease resistance [24,33]. SA/SAG also activates SAR and SA-responsive signaling factors, such as *PR1*, which play roles in plant defense [13,24,34]. A previous study suggested the connection between SA level and programmed cell death/ROS production [35]. The early induction of conjugated SA in Surpass400 and 01-23-2-1 suggested that early SA storage might slowly induce free SA to trigger defense responses, such as oxidative burst and cell death, by which the plant cells are able to stop the fungal cells during their initial development. On the other hand, Westar showed the accumulation of bound SA at 11 dpi. Similar to the late activation of SA/JA factors, the susceptible Westar genotype could not recognize the presence of fungus at the early stage and trigger SA-responsive signals in a timely manner. It was not until the fungus had reached its necrotrophic stage that the increase in SA storage/accumulation was initiated. The accumulation of stored SA seemed to synchronize with SA-related transcription factors in all three genotypes, suggesting that a large section of SA and its responsive signals came from the hydrolysis of stored (i.e., inactive) SA.

### 3.4. Unconventional Signaling Transductions Were Observed from qPCR Results

There was also an unusual co-operation between SA and ET/JA signaling observed in Surpass400–H75 8-1 and Westar–H75 8-1/H77 7-2 during the late stage of infection. According to the conventional hormonal signaling theory, SA and ET/JA have an antagonistic relationship [1]; however, Sašek et al. (2012) [13] also observed the co-expression of both SA- and ET-related genes in the case of incompatible interaction in the *B. napus*–*L. maculans* pathosystem. The SA-/ET-/JA-related genes showed a gradual increase in expression after the transitive time point [36], and the defense genes downstream of these three major hormones coincided in induction from the transitive time point (7 dpi) to the necrotrophic stage (11 dpi). Similar patterns have also been observed by Sašek et al. (2012) [13] and Becker et al. (2017) [12]. It seems that the early recognition of AvrLm proteins by the resistant genotypes caused the distinct onset patterns of certain genes between susceptible and resistant backgrounds, such as the genes in hormone signaling (i.e., *ICS1* and *PR1*) and sulfur metabolism (i.e., *APR* genes).

This type of co-operation has been observed in other studies. Sašek et al. (2012) [13] also found the earlier activation of SA and ET/JA factors after the inoculation of avirulent *L. maculans* isolate. Genes such as *WRKY70* (SA-responsive), *ACS2* (ET-responsive), and *CHI* (ET-/JA-responsive) were expressed in the case of resistant genotype (by incompatible interaction) before or at 7 dpi. Becker et al. (2017) [12] also observed the early activation (3 dpi) of SA-/ET-/JA-responsive genes from the resistant *B. napus* genotype. The co-expression of the factors from both SA/ET and JA aspects can be explained as a balanced general defense signaling strategy. SA and ET have been found to promote oxidative burst and lesion formation, while JA is able to reduce the effects of ROS-induced cell senescence [37,38]. As both susceptible (Westar–H75 8-1/H77 7-2) and intermediate (Surpass400–H75 8-1) cotyledons showed high induction of defense signaling around 11 dpi, the plant body may trigger innate balancing mechanisms to prevent the detrimental effects of excessive defense activities. Spoel et al. (2007) [11] postulated that the PCD induced by avirulent pathogen may attract the necrotrophs, and the activation of JA signaling is able to hinder the spread of necrotroph. From the situation of *L. maculans* infection, it is possible that the fungus would transit to necrotrophic stage when dead cells are formed by HR, the activation of JA may prevent the necrotrophic *L. maculans* among the dead tissues.

### 3.5. Two Isolates (HCRT75 8-1 and HCRT77 7-2) Induced Differential Patterns of Hormonal Gene Expression in Incompatible Interactions

Both Surpass400–H77 7-2 (AvrLm1—BLMR1/LepR3) and 01-23-2-1–H75 8-1/–H77 7-2 (AvrLm4-7/Rlm7) displayed total resistant phenotypes. The RT-qPCR results suggested that distinct expression profiles were observed among those genotypes.

It is possible that different versions of gene-for-gene interactions (i.e., Avr–R pairs) have distinct subsequent patterns of defense signaling cascades. In the gene-for-gene interaction between *Arabidopsis thaliana* and *Pseudomonas syringae*, Century et al. (1995) [39] found that RPS2-mediated resistance is largely repressed in *ndr1* mutant Arabidopsis lines, while RPM1-mediated resistance was partially suppressed under the same mutant background. Two Avr proteins reacting to one R protein also exert distinct defense signaling responses: both AvrRpt2 and AvrRpm1 caused a defense response towards RPM1; however, AvrRpt2 only resulted in a weak defense response, while AvrRpm1 was able to trigger the typical HR phenotype. Even the different versions of gene-for-gene interactions may exert different ways of signal transductions for some genes.

## 4. Materials and Methods

### 4.1. Plant Growth and Leptosphaeria maculans Isolates

Three *Brassica napus* genotypes Westar (no *Rlm* genes), Surpass400 (*BLMR1*/*LepR3* and *BLMR2*/*RlmS*), and 01-23-2-1 (*Rlm7*)) were grown in Sunshine Professional Growing Mix (SumGro Horticulture), with a cycle of 16 h of light (light intensity: 323 μmol/S·m^2^, 22 °C) and 8 h of night (16 °C) at 50–60% relative humidity. *L. maculans* isolates H75 8-1 (genotype: *avrLm1*, *AvrLm2*, *avrLm3*, *avrLm4*, *AvrLmJ1-5*, *AvrLm7*, *AvrLm6*, *avrLm9*, *AvrLm11*, *avrLepR1* and *AvrLepR2*) and H77 7-2 (genotype: *AvrLm1*, *avrLm2*, *avrLm3*, *AvrLm4*, *AvrLmJ1-5*, *AvrLm7*, *AvrLm6*, *avrLm9*, *AvrLm11*, *avrLepR1*, and *avrLepR2*) were cultured on V8 juice agar medium (Campbell’s, Camden, NJ, USA) at room temperature in the light. The culturing of isolates lasted for 10–14 days to produce pycnidiospores. Each culture was scraped and washed by 2 mL of distilled water to collect pycnidiospores.

### 4.2. Cotyledon Inoculation

The harvested pycnidiospores were adjusted to a concentration of 2 × 10^7^ spores/mL for cotyledon inoculation tests.

The cotyledons of seven-day-old seedlings were punctured by a modified tweezer and inoculated by 10 µL diluted inoculum. Each cotyledon lobe was punctured by a modified tweezer; thus, there were four points of inoculation on each seedling canola cotyledon.

### 4.3. Lesion Size Quantification

The cotyledons from 3 to 14 days post-inoculation (dpi) were scanned, and the lesion size was measured using the APS Assess 2.0 software (American Phytopathological Society, Saint Paul, MN, USA, 2008).

### 4.4. Trypan Blue Staining

Cotyledons (5, 7, and 11 dpi) were cut into 1 × 1 cm^2^ segments, immersed with 4 mL of clearing solution A (acetic acid/ethanol = 1:3, *v*/*v*), and shaken at a low speed overnight. Solution A was discarded, changed to clearing solution B (acetic acid/ethanol/glycerol = 1:5:1, *v*/*v*/*v*), and shaken at a low speed for at least 3 h. After the removal of clearing solution B, the cotyledons were stained with 2 mL of staining solution (0.01% trypan blue in lactoglycerol; lactic acid/glycerol/dH_2_O = 1:1:1, *v*/*v*/*v*) and shaken at a low speed overnight. The staining solution was changed to 60% glycerol as washing solution with low-speed shaking for at least 2 h. Finally, the washed cotyledon segments were ready to observe on clean slides. The staining experiment followed the protocol of Chung et al. (2006) [40].

### 4.5. Analysis of Bound Salicylic Acid (Bound SA)

The cotyledons at 3, 7, and 11 dpi were collected, lyophilized, and stored at −80 °C. The SA content from bound SA was released by acidic (HCl) hydrolysis. The levels of freed SA were measured by HPLC [33]. Both the control (water-inoculated) and inoculated cotyledons were measured in 3 biological replicates (0.1 g dry mass for each).

### 4.6. Gene Expression Analysis

Frozen cotyledons (1, 3, 5, 7, and 11 dpi) were ground in liquid nitrogen with a pestle and mortar. Total RNA was extracted with TRI reagent (Sigma-Aldrich, St. Louis, MO, USA). Total RNA was purified by DNaseI treatment with a recombinant DNaseI RNase-free kit (Millipore Sigma, Oakville, ON, Canada). Purified RNA (1 µg) was used to synthesize cDNA using the GOScript Reverse Transcription System (Promega, Madison, WI, USA). The cDNA stock solution was diluted to 100 ng/µL. Quantitative-PCR was performed by loading 1 µL of cDNA (100 ng) into the 10 µL reaction system of the IQ^TM^ SYBR^®^ Green Supermix (BioRad, Hercules, CA, USA). Experiments were based on three biological replicates.

The qPCR program used for all of the analyzed genes (except for *ACO1*) was 95 °C for 3 min; followed by 39 cycles of 95 °C for 15 s and 60 °C for 20 s; followed by a melting curve analysis.

As the qPCR for *ACO1* using the program mentioned above showed low quality, the qPCR program used for *ACO1* was 95 °C for 3 min; followed by 39 cycles of 95 °C for 15 s, 55 °C for 1 min, and 72 °C for 1 min; followed by a melting curve analysis.

All qPCR primers are listed in Supplementary Table S1. The relative level of gene expression was analyzed with the 2^−ΔΔCT^ method described by Livak and Schmittgen (2001) [41]. *Actin* was used as a reference gene to normalize the expression of the target genes.

### 4.7. Statistical Analysis

Unless specified, the analyses of samples used at least three biological replicates. The statistical analyses were performed using the Fisher’s least significant difference (LSD) method with the SAS 9.4 software. The Fisher’s LSD was applied to lesion test, gene expression, and bound SA measurement, in order to observe effectiveness of resistance in three genotypes when inoculated with two isolates.

## 5. Conclusions

In conclusion, this study showed that the regulation of hormonal signaling is crucial for plant defense in *B. napus* under the pressure of *L. maculans*. Different trade-off patterns for some hormonal responsive factors led to distinct levels of severity. Among them, SA-responsive factors were found to play pivotal roles in stronger resistance in *B. napus*, in which early SA signaling and subsequent SAR, such as *WRKY70* and *PR1*, possibly play a central role in the defense against *Brassica napus*. Compared with the incompatible interaction, the compatible interaction showed later activation of the SA-/JA-/ET-related genes studied in this research, suggesting that the late activation of massive defense signals may not rescue *B. napus* from *L. maculans* invasion. Again, it implicates the advantages of priming of defense activities in *B. napus* from more resistant genotypes (i.e., Surpass400 and 01-23-2-1). The distinct onset patterns of the hormone-responsive genes between these two types of interactions reflect the importance of early activation of essential defense genes to stem the early hyphal development of *L. maculans*. For future directions, the connection between hormone signaling and other defense mechanisms is suggested, such as reactive oxygen species (ROS) signaling and mitogen-activated protein kinase (MAPK) cascades. The interaction between different hormonal signaling pathways should also be another intriguing area to explore.

## Data Availability

The original contributions presented in the study are included in the article/supplementary materials, further inquiries can be directed to the corresponding author/s.

## Supplementary Materials

The following are available online at https://www.mdpi.com/article/10.3390/ijms22094714/s1.
